# Long Noncoding RNAs (lncRNAs) as Biomarkers of Prognosis in TNBC and Non-TNBC Breast Cancer Patients

**DOI:** 10.3390/ijms27177617

**Published:** 2026-08-25

**Authors:** Marcio Luis Acencio, Xinhui Wang, Flavia R. Rotea Mangone, Dirce Maria Carraro, Andrea S. Llera, Eliana S. F. W. Abdelhay, Nora Artagaveytia, Adrian Daneri-Navarro, Eduardo Moraes Reis, Leonardo Sanches, Roger Chammas, Osvaldo L. Podhajcer, Carlos Velazquez, Maria A. Nagai

**Affiliations:** 1 Bioinformatics Core Unit, Luxembourg Centre for Systems Biomedicine, University of Luxembourg, L-4362 Esch-sur-Alzette, Luxembourg; mlacencio@gmail.com (M.L.A.);; 2 Centro de Investigação Translacional em Oncologia—CTO, Instituto do Câncer do Estado de Sao Paulo (ICESP), Faculdade de Medicina da Universidade de São Paulo (FMUSP), Avenida Doutor Arnaldo, 251, 8° Floor, Sao Paulo 01246-000, SP, Brazil; flavia.mangone@hc.fm.usp.br (F.R.R.M.);; 3 Comprehensive Center for Precision Oncology, Universidade de Sao Paulo, Avenida Doutor Arnaldo, 251, 8° Floor, Sao Paulo 01246-000, SP, Brazil; emreis@iq.usp.br (E.M.R.);; 4 Clinical and Functional Group, Centro Internacional de Pesquisa (CIPE), AC Camargo Cancer Center, Sao Paulo 01508-010, SP, Brazil; 5 Molecular and Cellular Therapy Laboratory, Fundación Instituto Leloir-CONICET, Buenos Aires C1405BWE, Argentina; 6 Bone Marrow Transplantation Unit, Instituto Nacional de Câncer, Rio de Janeiro 20230-130, RJ, Brazil; eabdelhay@inca.gov.br; 7 Hospital de Clínicas, Facultad de Medicina, Universidad de la República, Montevideo 11600, Uruguay; nartagave@fmed.edu.uy; 8 Department of Cellular and Molecular Biology, Universidad de Guadalajara, Guadalajara 44430, Mexico; 9 Department of Biochemistry, Institute of Chemistry, Universidade de Sao Paulo, Sao Paulo 05508-000, SP, Brazil; 10 Universidad de Sonora, Hermosillo 83000, Mexico

**Keywords:** breast cancer, lncRNAs, *PART1*, *VLDLR-AS1*, *EGOT*, prognostic, biomarker

## Abstract

Functional studies have shown that long noncoding RNAs (lncRNAs) play different roles in gene expression regulation, acting as epigenetic factors. Accumulating evidence indicates that lncRNAs modulate diverse biological processes, and their altered expression is associated with the development and progression of cancer, including BC. Here, aiming to identify lncRNAs associated with breast cancer, we performed a re-analysis of the expression data from 1071 tumors of eligible patients in the LACRN-MPBC Study. Using normalized data, we compared the expression profiles of triple-negative breast cancer (TNBC; 170 cases) and a heterogeneous group of non-TNBC tumors (782 cases). A total of 59 differentially expressed lncRNAs (DELncRNAs) could be identified based on the criteria of |log2FC| ≥ 1 and FDR < 0.05, using the DESeq2 R package. In the Kaplan–Meier survival analysis, we identified DELncRNAs that affect disease-free survival and/or overall survival, with eight of them in TNBC and 27 in non-TNBC patients. Multivariate Cox proportional-hazards models were used to evaluate independent predictors of survival. Two DELncRNAs, *VLDLR-AS1* and *PART1*, were identified as independent prognostic markers for TNBC patients, while seven (*LINC00239*, *FAM30A*, *LINC00842*, *LINC01315*, *EGOT*, *LY6E-DT*, and *SLC25A21-AS1*) were independent prognostic markers for non-TNBC patients. Our computational study identifies several candidate lncRNAs associated with clinical outcomes in breast cancer. However, the DELncRNAs identified here should be interpreted as preliminary candidates, which require future validation and functional studies to determine their biological roles and evaluate their potential as prognostic biomarkers.

## 1. Introduction

Breast cancer is the most common neoplasm affecting women worldwide, being a global public health problem [1]. Based on GLOBOCAN data, it is estimated that 2.3 million new cases and 670,000 breast cancer deaths occurred in 2022. If these estimates remain, it can be estimated that 3.2 million new cases and 1.1 million breast cancer-related deaths will occur in 2050, representing increases of 38% and 68%, respectively [2].

Breast cancer is a heterogeneous disease, and based on expression profiling, such as the PAM50 gene signature, breast cancer is classified into four distinct molecular subtypes, including luminal A, luminal B, HER2-enriched, and triple-negative [3]. Moreover, the St. Gallen Consensus established the surrogate subtypes of breast cancer, based on the immunohistochemical of biomarkers markers, including the steroid receptors, estrogen receptor (ER) and progesterone receptor (PR), overexpression of HER2 oncoprotein, and on the expression of the cell proliferative index (Ki-67), classifying them as Luminal A (ER+/PR+/HER2−/Ki-67 low); Luminal B (ER+/PR+/HER2−/+/Ki-67 high); HER2 overexpression (ER−/PR−/HER2+), and triple-negative breast cancers/TNBCs (ER−/PR−/HER2−) [4]. TNBCs account for 15–20% of all breast cancer cases and comprise a highly heterogeneous subset of breast tumors characterized by high aggressiveness and invasiveness, with the worst outcome [5]. There are still no specific successful therapeutic strategies for the treatment of TNBC patients, posing an important area of investigation in breast cancer [6]. The molecular and immunophenotypic classification is important for treatment strategy decisions and to better tailor patient outcomes [7]. However, some patients still face intrinsic or acquired treatment resistance, leading to mortality.

Despite the advances in breast cancer molecular classification, efforts are still needed to identify new prognostic and predictive biomarkers of therapeutic response for breast cancer. Only 2% of the human genome consists of protein-coding genes. Therefore, approximately 90% of the remaining genome is transcribed in non-protein-coding transcripts, which are classified as noncoding RNAs [8,9]. The noncoding RNAs (ncRNAs) are classified as short ncRNAs (miRNAs, siRNAs, and piRNAs) and long ncRNAs (long noncoding RNAs; lncRNAs) [10]. Historically, lncRNAs have been defined as ncRNAs longer than 200 nucleotides; however, more recently, a Consensus Statement publication from 2023 recommends that lncRNAs should be considered molecules longer than 500 nucleotides [11]. LncRNAs are widely expressed in different tissues and several studies indicate that lncRNAs play an essential role in different biological processes and in the pathophysiology of cancer [12]. Accumulating evidence in the literature indicates that lncRNAs act at the transcriptional, post-transcriptional, and epigenetic modification levels [13]. LncRNAs can directly influence gene expression, regulating the activity of promoters, acting in chromatin remodeling, and in the recruitment of epigenetic factors [10]. Altered expression of different lncRNAs has been found in breast cancer, being associated with increased cell proliferation, inhibition of apoptosis, epithelial–mesenchymal transition, invasiveness, metastasis formation, poor prognosis, and drug resistance in breast cancer [14]. Therefore, investigating lncRNAs associated with breast cancer tumorigenesis is essential for identifying potential new biomarkers that improve molecular classification, prognostication, and prediction of drug response.

In this study, aiming to identify lncRNAs associated with breast cancer prognosis, we performed a reanalysis of the gene expression data generated using cDNA microarrays in the Molecular Profile of Breast Cancer Study (MPBCS) cohort from the US-LACRN (US-Latin America Cancer Research Network) [15]. We have identified 59 differentially expressed lncRNAs (DELncRNAs) in the comparison between triple-negative breast cancer (TNBC) and a heterogeneous group of non-TNBC breast tumors. Furthermore, we assessed the prognostic impact of these DELncRNAs on overall survival and disease-free survival. Here, we identified a set of lncRNAs as potential prognostic candidate biomarkers for breast cancer; however, our work should be considered a discovery study that provides important information for future clinical and experimental validation.

## 2. Results

### 2.1. Identification of Differentially Expressed lncRNAs (DELncRNAs)

**Figure 1:**
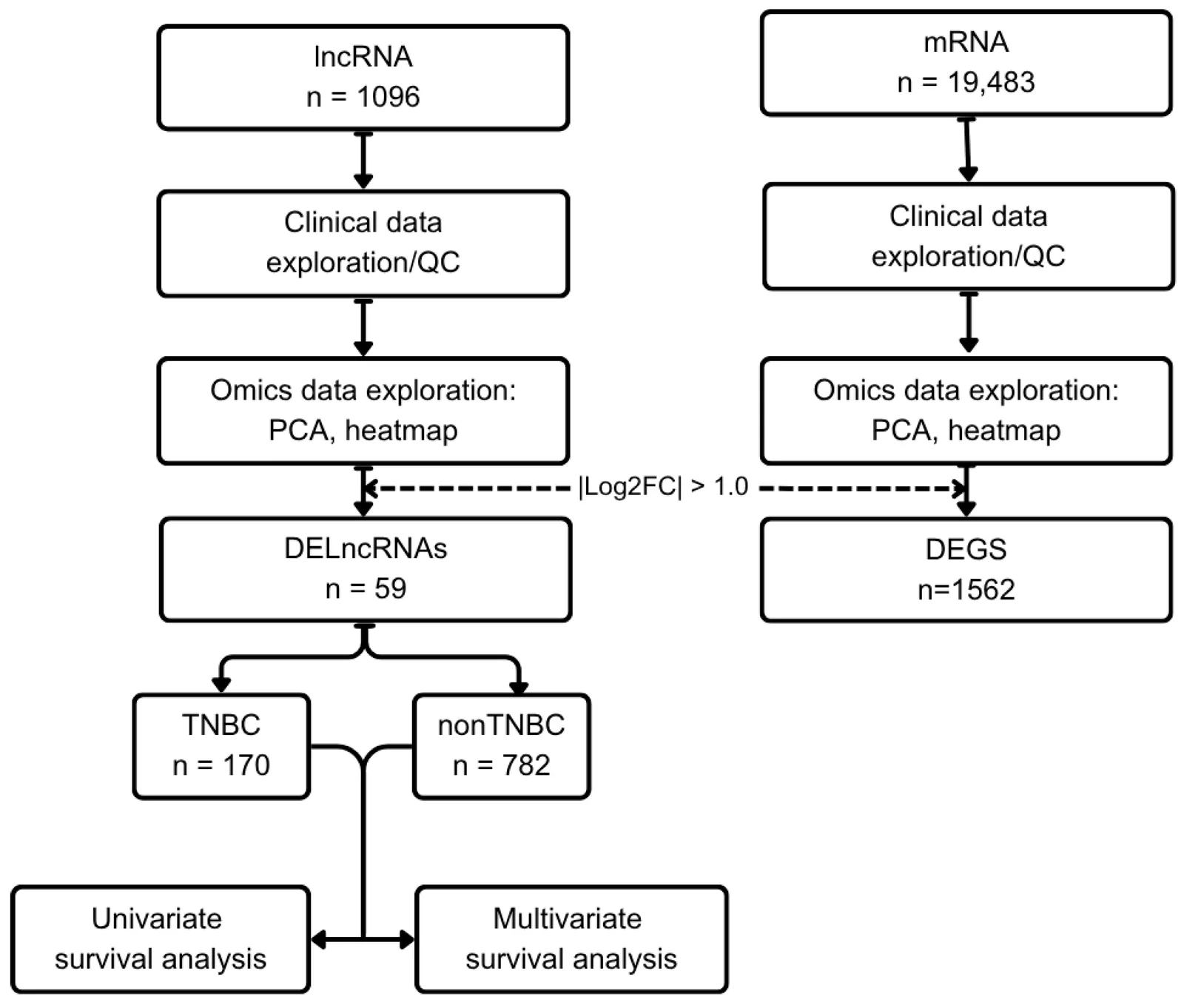
Flowchart summarizing the data analysis for identifying differentially expressed long non-coding RNAs (DELncRNAs) in breast cancer. This schematic illustrates the overall study design. Normalized long non-coding RNA (lncRNA) and messenger RNA (mRNA) expression data, together with corresponding clinical information, were used for exploratory and differential expression analyses. Samples were stratified into triple-negative breast cancer (TNBC) and non-TNBC groups for comparative evaluation. Arrows indicate the sequence of analysis.

**Figure 2:**
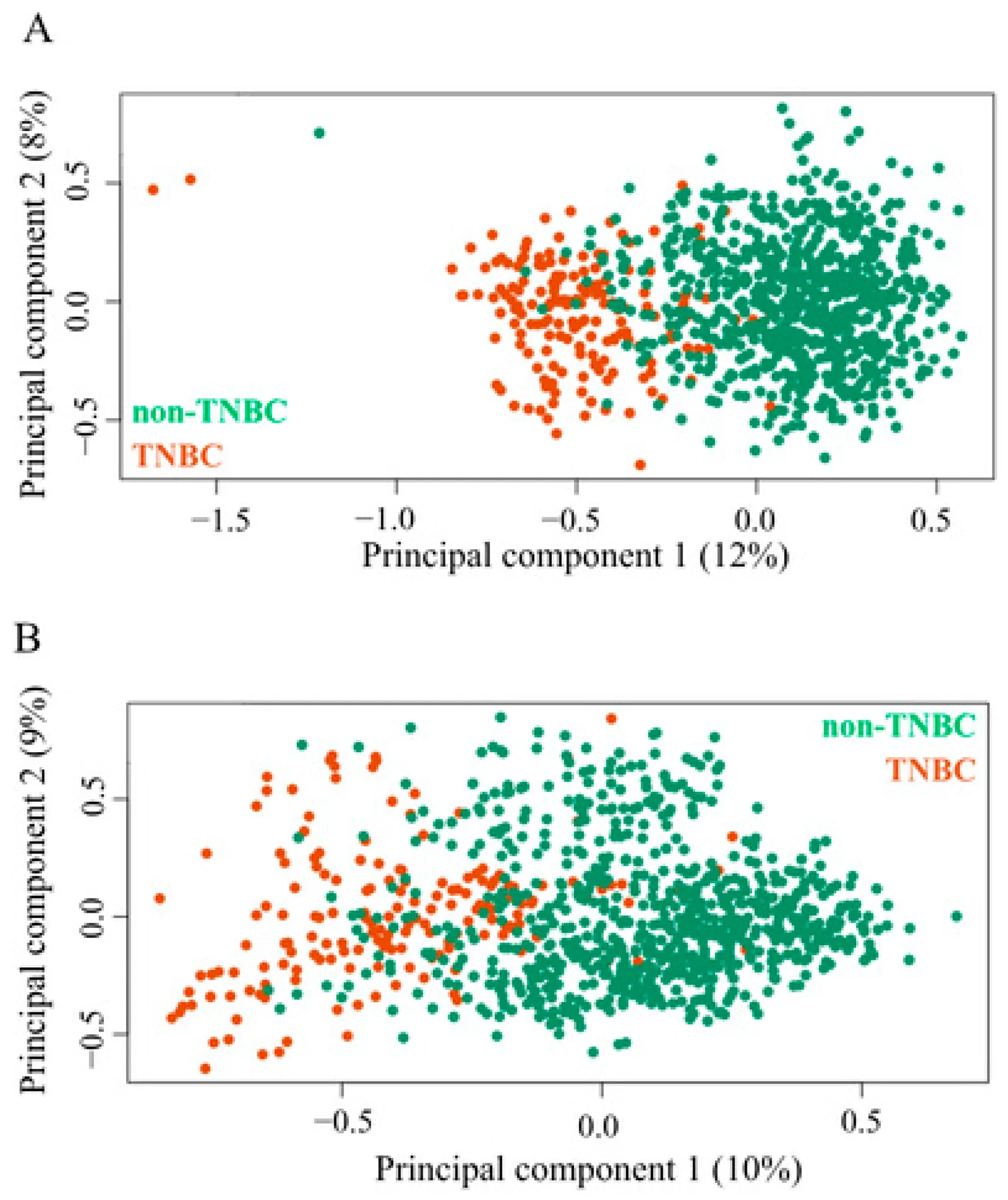
Principal component analysis (PCA) of mRNA and lncRNA expression profiles in breast cancer samples. PCA using normalized expression data from 952 breast cancer samples was used to assess global transcriptomic variation between TNBC and non-TNBC groups. (**A**) mRNA-based PCA (19,483 probe sets). (**B**) lncRNA-based PCA (1096 lncRNAs). Each point represents a sample, colored by subtype. Axis percentages indicate variance explained by the first two principal components.

**Figure 3:**
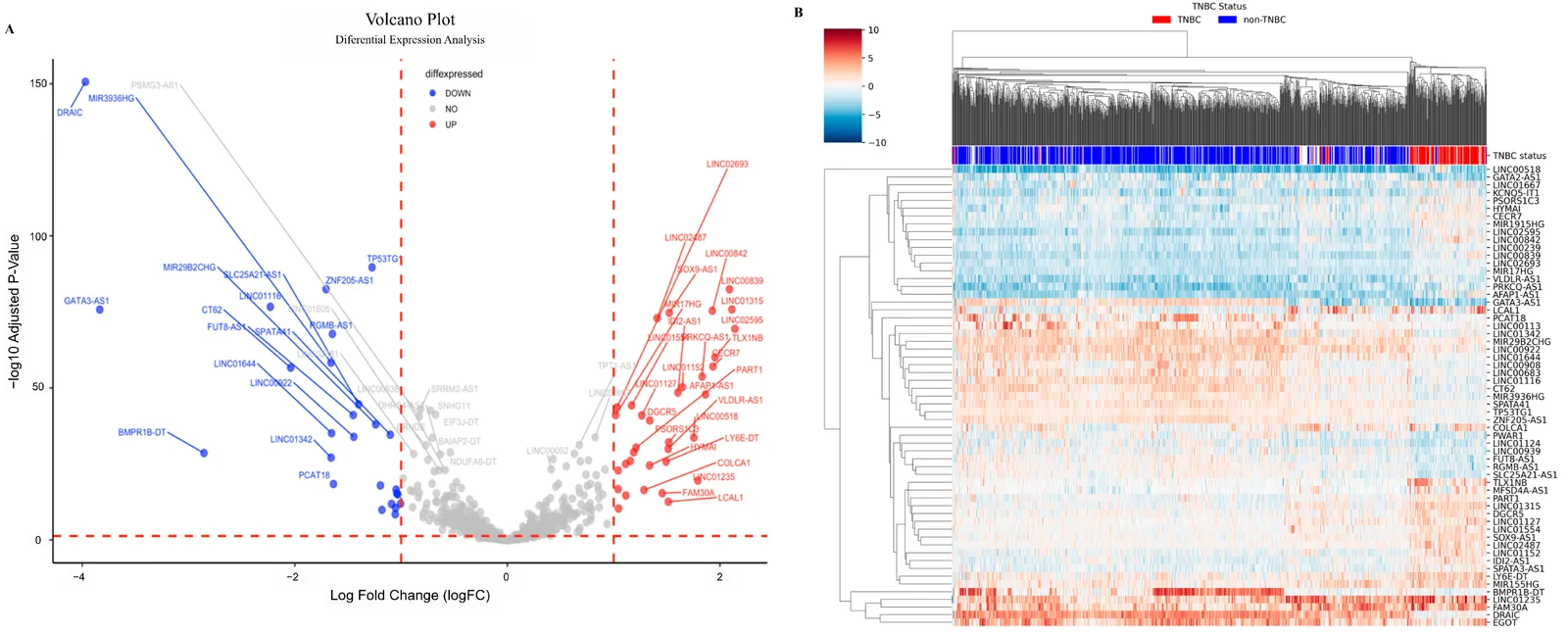
Differential expression analysis of lncRNAs between TNBC and non-TNBC samples. (**A**) Volcano plot showing the 59 differentially expressed lncRNAs identified between TNBC and non-TNBC groups. Each point represents one lncRNA. The x-axis indicates log_2_ fold change (log_2_FC), and the y-axis shows—log_10_ adjusted *p*-value. Upregulated lncRNAs in TNBC are shown in red, downregulated lncRNAs in blue, and non-significant lncRNAs in gray. (**B**) Heatmap of the 59 differentially expressed lncRNAs based on hierarchical clustering of normalized expression values. Columns represent individual samples and rows represent lncRNAs. Samples are grouped according to TNBC status, and the color scale reflects relative expression levels.

Aiming to identify long non-coding RNAs (lncRNAs) associated with breast cancer, we performed a reanalysis of expression data from 1071 tumors from eligible patients in the LACRN MPBCS project. The study flowchart is presented in Figure 1. The distribution of the demographic and clinicopathological parameters is shown in Table S1. We classified the tumors into different subtypes using PAM50 gene signatures. Principal component analysis (PCA), based on similarities and dissimilarities in the expression profiles of mRNAs and lncRNAs, distinguished between triple-negative breast cancer (TNBC) and non-TNBC tumors (Figure 2). Next, we used normalized data to compare expression profiles between TNBC (170 cases) and non-TNBC (782 cases) tumors. A total of 59 (34 up-regulated and 25 down-regulated) DELncRNA could be identified based on the criteria of |log_2_FC| > 1 and FDR < 0.05, using the DESeq2 R package. Figure 3A and Figure 3B present the volcano plot and heatmap, respectively, illustrating the distribution of the 59 DELncRNAs identified in the comparison between TNBC and non-TNBC.

### 2.2. Validation of the DELncRNAs Expression in the TCGA and METRABIC Databases

Although we have not yet validated the expression results of DElncRNAs and their impact in an independent population of Latin American women, we performed a validation to assess whether the DElncRNAs identified here can distinguish TNBC from non-TNBC phenotypes in the TCGA and METRABIC datasets. Only 35 of the DElncRNAs we identified were present in the TCGA, and 21 were present in the METRABIC dataset; however, the results confirmed that the expression direction of these lncRNAs was similar to that observed in our discovery population and that they were able to separate TNBC tumors from non-TNBC tumors (Figure S1). We found that the fold changes for those lncRNAs were greater in our population than in the TCGA dataset.

### 2.3. Impact of the DELncRNAs on Patients’ Survival

**Figure 4:**
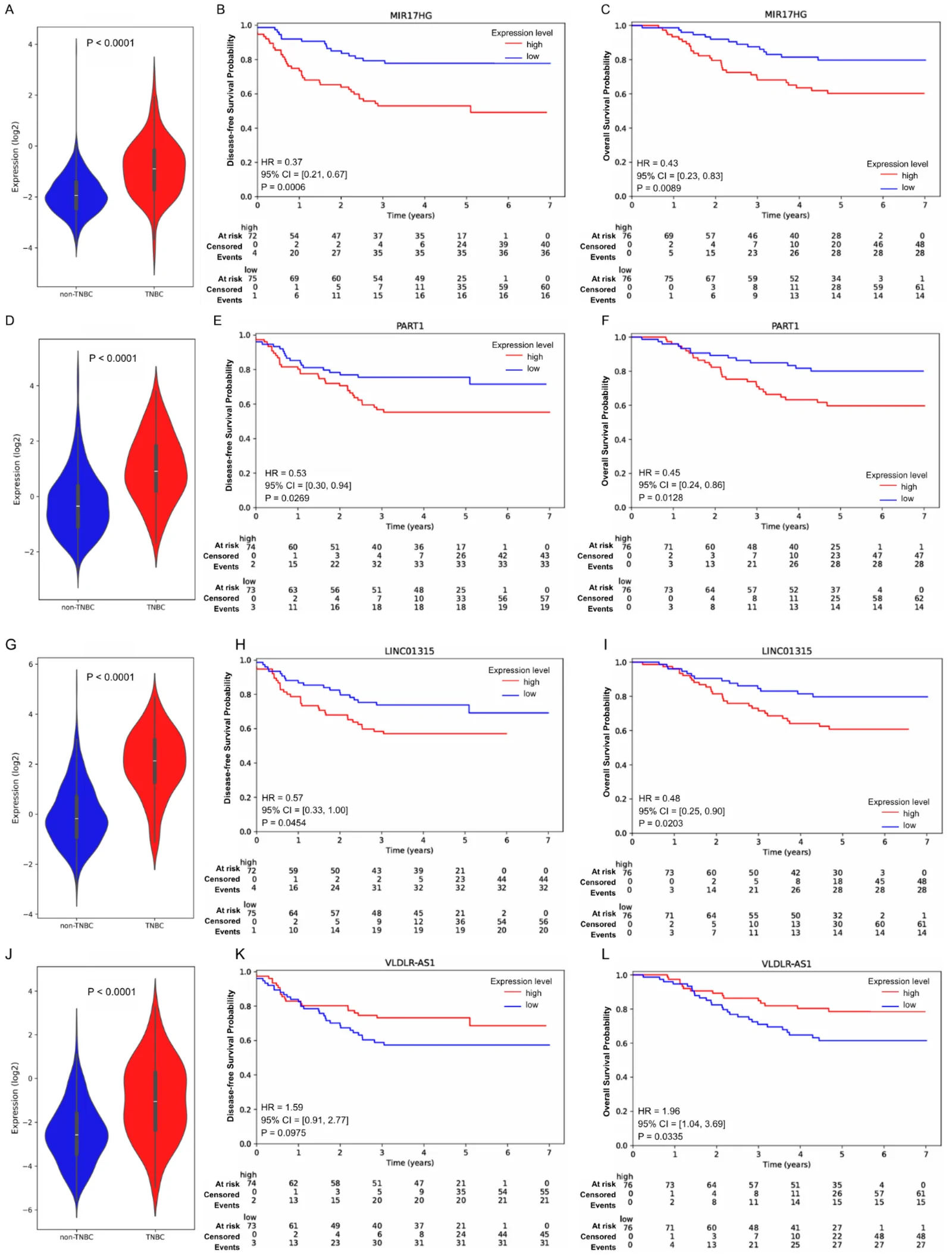
Expression levels and prognostic impact of 4 DELncRNAs in TNBC. Violin plots of 4 DELncRNAs expression in TNBC tumors: *MIR17HG* (**A**); *PART1* (**D**); *LINC01315* (**G**); *VLDLR-AS1* (**J**). Kaplan–Meier disease-free survival (DFS) and overall survival (OS) analysis of TNBC patients stratified by the expression of the lncRNAs *MIR17HG* ((**B**), DFS; (**C**), OS); *PART1* ((**E**), DFS; (**F**), OS); *LINC01315* ((**H**), DFS; (**I**), OS); *VLDLR-AS1* ((**K**), DFS; (**L**), OS). Hazard ratios were estimated using Cox proportional hazards regression. High expression was used as the reference. Statistical significance was set at *p* < 0.05.

We performed univariate and multivariate Cox regression analyses to assess the potential prognostic value of the 59 DELncRNAs identified in the TNBC vs. non-TNBC comparison. The Kaplan–Meier survival curves show that eight of these DELncRNAs were associated with disease-free survival (DFS) and/or overall survival (OS) in TNBC patients (Figure 4 and Table S2). Both DFS and OS rates in patients with tumors showing high expression of *MIR17HG*, *PART1*, and *LINC01315* were significantly shorter than those in patients with tumors showing low expression of these DELncRNAs. In addition, high expression of *DGCR5* and low expression of *LINC00239* and *PRKCQ-AS1* were associated with worse DFS only, and low expression of *VLDLR-AS1* and *EGOT* were significantly associated with worse OS only.

**Figure 5:**
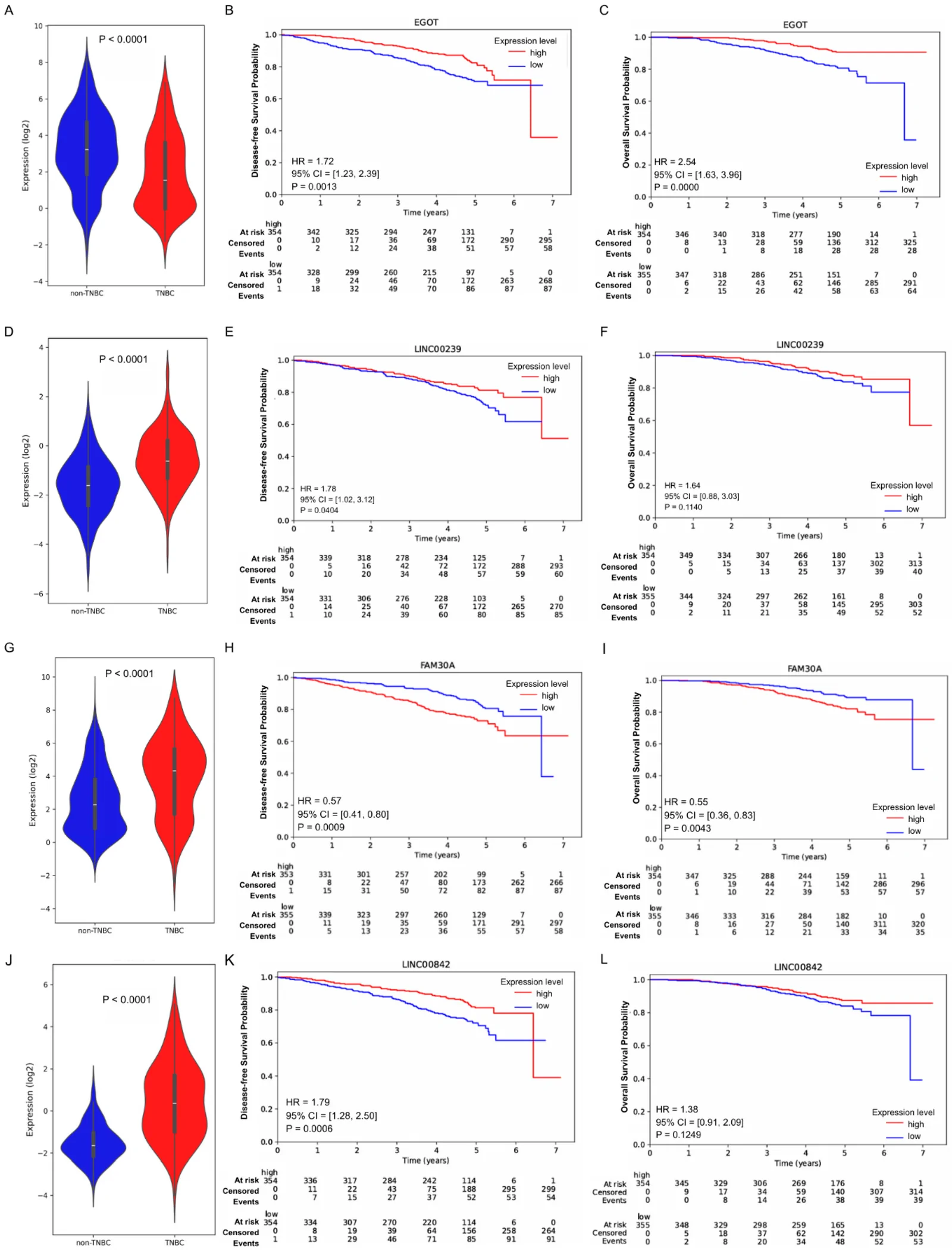
Expression levels and prognostic impact of 4 DELncRNAs in non-TNBC. Violin plots of 4 DELncRNAs expression in TNBC tumors and non-TNBC tumors: *EGOT* (**A**); *LINC00239* (**D**); *FAM30A* (**G**); *LINC00842* (**J**). Kaplan–Meier disease-free survival (DFS) and overall survival (OS) analysis of the TNBC patients stratified by the expression of the lncRNAs EGOT ((**B**), DFS; (**C**), OS); *LINC00239* ((**E**), DFS; (**F**), OS); *FAM30A* ((**H**), DFS; (**I**), OS); *LINC00842* ((**K**), DFS; (**L**), OS). Hazard ratios were estimated using Cox proportional hazards regression. High expression was used as the reference. Statistical significance was set at *p* < 0.05.

Twenty-seven out of the 59 DELncRNAs showed prognostic value for patients with non-TNBC (Figure 5 and Table S3). Twelve of these DELncRNAs showed impact in both DFS and OS, low expression of 9 DELncRNAs (*TP53TG1*, *ZNF205-AS1*, *GATA3-AS1*, *FUT8-AS1*, *SPATA41*, *BMPR1B-DT*, *LINC00908*, *LINC01124*, and *EGOT*) and high expression of 3 DELncRNAs (*IDI2-AS1*, *FAM30A*, and *LCAL1*), were associated with worse DFS and OS in patients with tumors of non-TNBC. We also found 8 DELncRNAs associated only with DFS rates, including high expression of *MIR17HG* and *MIR1915HG* and low expression of *LINC01315, LINC00842*, *MIR3936HG*, *CT62*, *LINC00239*, and *PCAT18*, which were associated with poor prognosis. In addition, 7 DELncRNAs were found to affect OS only. Patients with non-TNBC tumors showing high expression of *LINC01127*, *LY6E-DT*, or low expression of *LINC00683*, *LINC01152*, *SLC25A21-AS1*, *MIR29B2CHG*, and *LINC01644* had poor outcomes.

**Figure 6:**
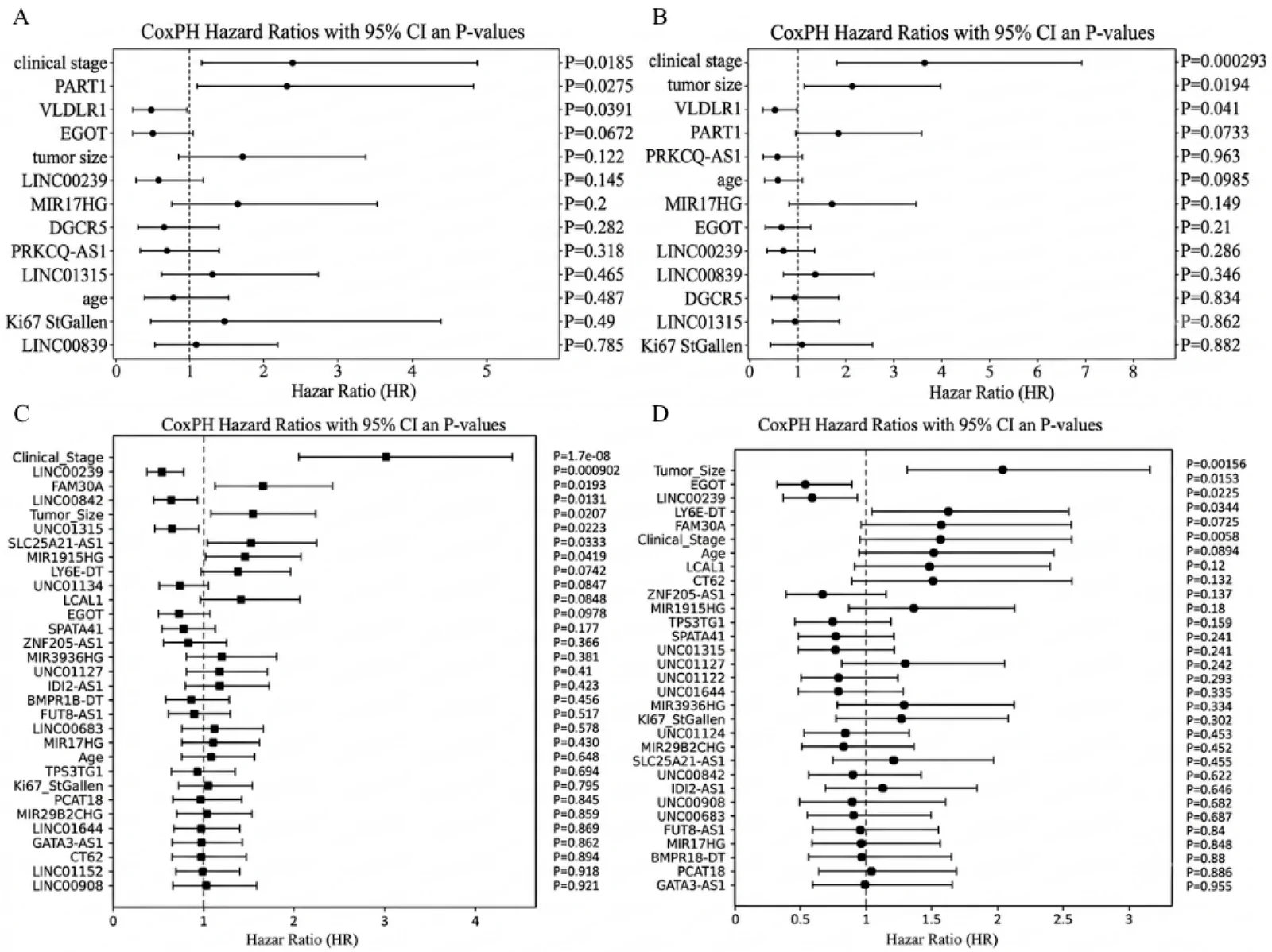
Multivariate Cox regression analysis of clinicopathological variables and DELncRNAs. Forest plots show hazard ratios (HRs) with 95% confidence intervals (CIs) and *p*-values for overall survival (OS) and disease-free survival (DFS). Multivariate Cox regression analyses for OS (**A**,**C**) and DFS (**B**,**D**) in TNBC and non-TNBC patients, respectively. The vertical dashed line indicates HR = 1. Variables with HR > 1 indicate increased risk, whereas those with HR < 1 indicate a protective effect.

Further, we used multivariate Cox regression to identify which DELncRNAs associated with TNBC or non-TNBC could serve as independent prognostic factors for breast cancer. The results showed that nine DELncRNAs (*LINC00239*, *FAM30A*, *LINC00842*, *LINC01315*, *MIR1915HG*, *SLC25A21-AS1*, *LY6E-DT*, and EGOT) and 2 DELncRNAs (*PART1* and *VLDLR-AS1*) in non-TNBC and TNBC patients, respectively, were independent prognostic factors (Figure 6).

### 2.4. DELncRNAs Causal Interaction Network and Functional Enrichment Analysis

**Figure 7:**
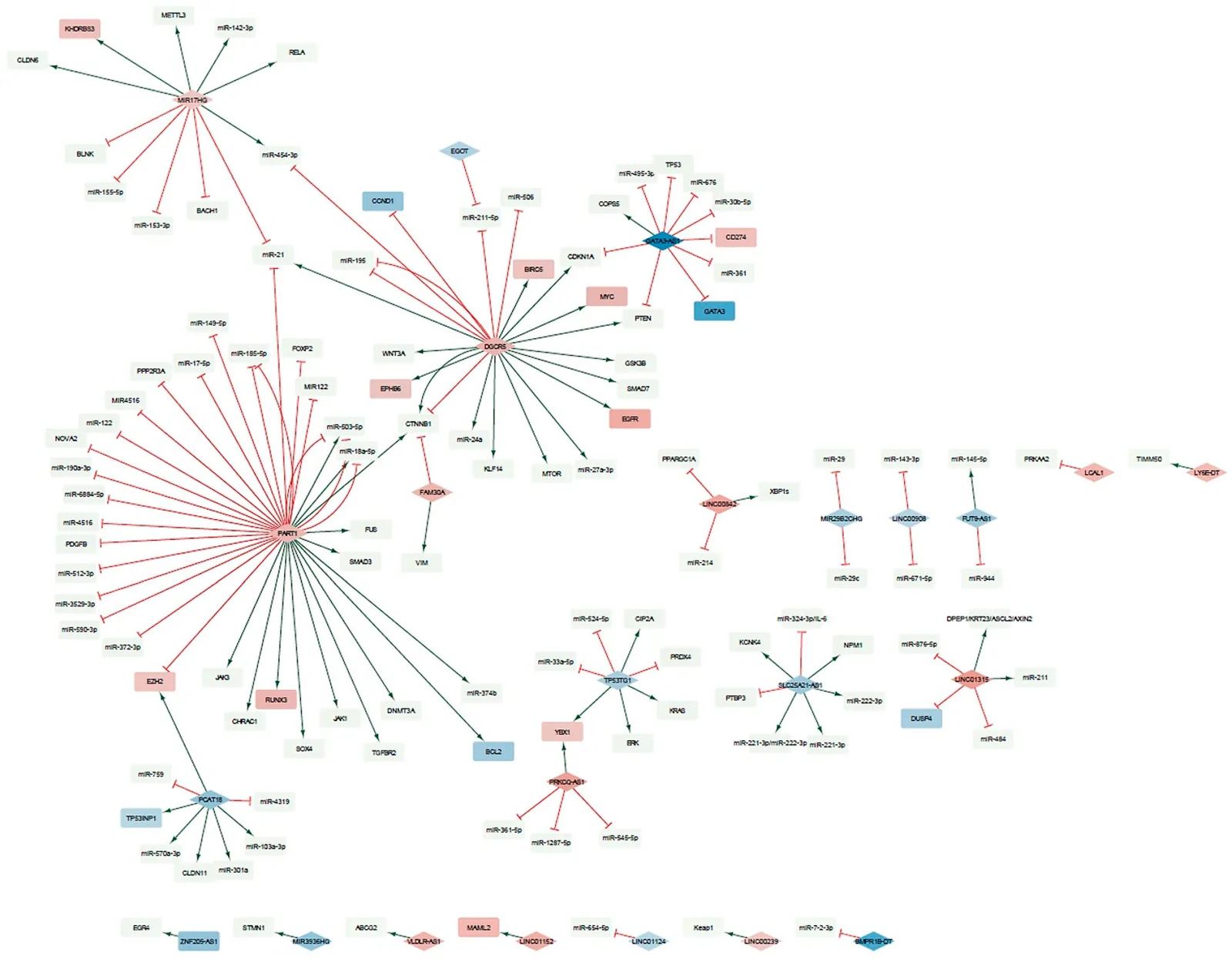
Interaction network of DFS- and OS-associated DELncRNAs. Causal interaction network of DELncRNAs associated with disease-free survival (DFS) and overall survival (OS). This network was constructed using experimentally validated interactions from the RNAInter database. Diamond-shaped nodes represent DELncRNAs. Rectangular nodes represent interacting proteins or miRNAs. Node color follows a blue-to-red gradient according to log_2_ fold change (log_2_FC), where blue indicates downregulation and red indicates upregulation. Light green nodes represent interacting proteins or miRNAs without available expression data or proteins encoded by non-differentially expressed genes (non-DEGs). Green arrows indicate activation, while red T-headed lines indicate inhibition.

Experimentally validated causal interactions between lncRNAs and proteins and miRNAs taken from the RNAInter database (confidence score > 0.5) were identified for 24 of the 32 DFS- and OS-associated DELncRNAs. These interactions were used to construct the survival-associated DELncRNAs interaction network (Figure 7). The resulting network consisted of a large component containing seven DELncRNAs (*MIR17HG*, *GATA3-AS1*, *EGOT*, *DGCR5*, *FAM30A*, *PART1*, and *PCAT18*) connected to 41 proteins and 34 miRNAs through 83 interactions, a smaller subnetwork centered on *TP53TG1* and *PRKCQ-AS1*, and 15 isolated DELncRNA-centered subnetworks. Within the large component, five distinct modules centered on *MIR17HG*, *GATA3-AS1*, *DGCR5*, *PART1*, and *PCAT18* are evident. *FAM30A* and *EGOT*, on the other hand, are more peripheral, with *FAM30A* connected to the giant component via CTNNB1, and *EGOT* connected through miR-211-5p. Inter-module communication in the giant component consists of CDKN1A, PTEN, CTNNB1, EZH2, miR-21, and miR-454-3p. These shared mediators are known to be involved in cell proliferation, cell cycle control, and epigenetic regulation. To further contextualize the interaction network, functional enrichment analysis was performed on all protein-coding genes associated with the 24 survival-associated DELncRNAs. The top enriched biological processes are dominated by primary metabolic processes and immune-related pathways, including response to cytokine, cellular response to cytokine stimulus, and adaptive immune response (Figure S2). This enrichment profile indicates that the survival-associated DELncRNAs-interacting proteins are mainly involved in immune signaling and metabolic regulation rather than exclusively in canonical oncogenic cascades. These results are consistent with the network topology, in which several centrally positioned mediators, such as RELA, JAK1, JAK3, MTOR, and CTNNB1, are known coordinators of inflammatory and metabolic responses.

## 3. Discussion

Here, we conducted a computational discovery study to identify potential new candidate biomarkers by reanalyzing cDNA microarray data from a cohort of 1071 tumors from eligible breast cancer patients in the LACRN-MPBCS project [15]. Although lncRNAs do not encode proteins, they play a cellular role in regulating transcription and thus regulating different biological processes [12]. Altered expression of lncRNAs is well documented in cancer, including breast cancer [14]. Therefore, exploring the role of lncRNAs and their potential prognostic and predictive value for treatment response may have important clinical implications.

In this study, PCA of the lncRNAs on the cDNA microarray slide showed that they could differentiate TNBC from the heterogeneous group of non-TNBC patients. We then compared TNBC and non-TNBC patients and identified a set of DELncRNAs. We performed a comprehensive analysis of the role of these lncRNAs in patient survival using univariate analysis and multivariate Cox regression, identifying a subgroup of DELncRNAs that impact the prognosis of patients with TNBC and non-TNBC. We identified DELncRNAs as independent biomarkers that better predict prognosis for TNBC and non-TNBC patients. Among these DELncRNAs, five demonstrated potential to predict DFS (*LINC00239*, *LINC01315*, *LINC00842*, *FAM30A*, *SLC25A21-AS1*) and three to accurately predict OS (*LINC00239*, *EGOT*, *LY6E-DT*) in non-TNBC patients. Most of the identified DELncRNAs remain poorly characterized. Some studies have associated altered expression of these genes with tumorigenesis; however, evidence in breast cancer is still limited. Among these DELncRNAs, we highlight *LINC00239*, *LINC01315*, and *EGOT*. For instance, overexpression of *LINC00239* has been associated with colorectal, hepatocellular, esophageal, and renal carcinoma [16,17,18,19]. The oncogenic effects of *LINC00239* on the progression of colorectal carcinoma were associated with its suppressive action on ferroptosis, where *LINC00239* expression promotes CRC proliferation by interacting with Kelch-like ECH-associated protein 1 (Keap1), causing instability of the Keap1/Nrf2 complex [16]. In ESCC, *LINC00239* acts through the MBP-1/c-Myc axis to activate the EMT process [18] and by activation of the PI3K/AKT/mTOR pathway in acute myeloid leukemia [20]. Interestingly, in this study, we found that low expression of *LINC00239* was a strong predictor of survival in non-TNBC patients. However, we did not identify any studies in the literature associating *LINC00239* expression with breast cancer. The Keap1/Nrf2 and PI3K/AKT/mTOR pathways regulate proliferation and influence the resistance of breast cancer cells to chemotherapy and radiotherapy. These data justify further clinical and experimental studies to evaluate the role of *LINC00239* in breast cancer.

In our study, we identified lower expression of the lncRNA *LINC01315* in non-TNBC tumors compared with TNBC. Downregulation and upregulation of the lncRNA *LINC01315* have been identified in different types of tumors, including breast cancer. In gastric cancer, *LINC01315* was identified in a pyroptosis-related gene signature, and low *LINC01315* expression was associated with a worse prognosis in patients [21]. In colorectal cancer, high expression of *LINC01315* was identified in tissues and colon cancer cell lines, and *LINC01315* expression increases proliferation and migration in colon cancer cells [22]. A study identified *LINC01315* as regulated by METTL3 in an m6A-dependent pathway and showed higher levels in hepatocellular carcinomas compared to normal tissue [23]. These authors conducted functional studies that showed that *LINC01315* increases proliferation, migration, and invasion in hepatocellular carcinoma cells via the LINC01315/miR-185 5p/β-catenin/WNT signaling axis [23]. Using data from different studies, meta-analyses, and machine learning feature selection methods, Naorem LD et al. (2020) identified low expression of LINC01315 associated with worse prognosis in patients with TNBC [24]. Xue J et al. (2021) evaluated *LINC01315* expression by qPCR in 287 breast tumors and identified higher levels of *LINC01315* expression in luminal A and HER2+ subtype tumors than in luminal B and basal subtype tumors, and identified high expression of this lncRNA associated with a worse prognosis for patients with breast cancer of all subtypes [25]. Xiu Y et al. (2022) [26] demonstrated that *LINC01315* overexpression is associated with poorer survival in patients with TNBC subtype tumors. These authors showed that silencing *LINC01315* in MDA-MB-231 and BT-549 cells inhibits cell proliferation, migration, and invasion by modulating the expression of miR-876-5p/GRK5 [26]. Interestingly, our data show a possible dual effect of *LINC01315* in breast cancer, with high expression of *LINC01315* being associated with a worse prognosis for patients with TNBC tumors and low expression associated with a worse prognosis for patients with non-TNBC. However, only low *LINC01315* expression was an independent prognostic factor in non-TNBC. *LINC01315* expression is a potential candidate biomarker in breast cancer; however, its prognostic and predictive value for therapeutic response and its mechanism of action still need to be validated.

The expression of lncRNA *EGOT* (eosinophil granule ontogeny transcript; 3p26.1) has been associated with the progression of various tumor types, including breast cancer. High *EGOT* expression has been associated with tumor progression and a worse prognosis in gastric cancer, and functional studies indicate that its expression promotes proliferation [27]. In head and neck squamous cell carcinomas, *EGOT* expression varies with disease stage, and low expression is associated with a worse prognosis [28]. Low *EGOT* expression has been demonstrated in gliomas relative to adjacent non-tumor tissue, and *EGOT* overexpression inhibits proliferation and migration and induces apoptosis in U87 and U251 glioblastoma cells [29]. Xu SP et al. (2015) demonstrated for the first time an association between low levels of *EGOT* expression as an independent prognostic factor for poor overall survival in breast cancer patients [30]. Further, studies using publicly available datasets have found that low *EGOT* expression in breast cancer is associated with a poor prognosis in a genomic instability-related lncRNA model for predicting prognosis [31] and in a prognostic signature based on N7-Methylguanosine-Related lncRNAs [32]. More recently, Castro-Oropeza R et al. (2025) identified an association between low expression of lncRNA *EGOT* and poor overall survival in Mexican patients with breast cancer [33]. Our data corroborate these studies in breast cancer and reinforce the value of *EGOT* expression as a candidate prognostic biomarker in breast cancer.

In TNBC, we identified eight lncRNAs associated with patient survival in univariate analysis: *MIR17HG*, *PART1*, *LINC01315*, *DGCR5*, *LINC00239*, *PRKCQ-AS1*, *VLDLR-AS1*, and *EGOT*. However, in multivariate analysis, only the lncRNAs *PART1* and *VLDLR-AS1* were independent prognostic markers. We found that high expression of *PART1* and low expression of *VLDLR-AS1* contribute to a more effective identification of patients with a worse prognosis. The lncRNA *VLDLR-AS1* (Very low-density lipoprotein receptor-antisense 1; also known as *linc-VLDLR*) is located on chromosome 9p24.2 and is organized as an antisense transcript on the opposite strand of the *VLDLR* gene (NCBI database). *VLDLR-AS1* was found to be overexpressed in malignant hepatocytes, and exposure of HCC cells to different anti-cancer agents increases *VLDLR-AS1* intracellular expression and expression within extracellular vesicles (EVs) released by these cells [34]. *VLDLR-AS1* expression has been shown to be an independent predictor of recurrence risk in patients with thymomas [35]. Increased expression of *VLDLR-AS1* and *ABCG2* has been demonstrated in esophageal cancer, and *VLDLR-AS1* transported by extracellular vesicles (EVs) released by chemotherapeutic-resistant esophageal carcinoma cells can induce drug resistance through *ABCG2* expression in these cells [36]. More recently, reduced expression of *VLDLR-AS1* was identified in sporadic parathyroid adenomas [37], and increased expression of *VLDLR-AS1* was observed in hypoxia-exposed lung cancer cells [38]. To date, the functional role of *VLDLR-AS1* is poorly understood, and we have not identified any breast cancer studies. Further, clinical or functional studies to better understand its role are warranted, especially in TNBC.

Altered expression of *PART1* (prostate androgen-regulated transcript 1) has been identified in several pathologies and cancers, with a dual role in tumorigenesis, acting as either an oncogene or a tumor suppressor depending on the tumor type [39,40]. As reviewed by Ghafouri-Fard S et al. (2023), functional cancer studies show that *PART1* expression modulates biological processes such as proliferation, migration, invasion, and cell death through signaling pathways including PI3K/AKT, TLR, Wnt/β-catenin, and JAK/STAT3 [40]. In breast cancer, Jiang Z et al. (2020), using RNA-seq data and miRNA-seq data of luminal A breast cancer in The Cancer Genome Atlas (TCGA) database, identified an association between high expression of *PART1* and poor overall survival for breast cancer patients with tumors of the luminal subtype [41]. *PART1* overexpression was found to be associated with male breast cancer progression, and its knockdown in MCF7 and BT-20 breast cancer cells leads to reduced cell proliferation and invasion [42]. These authors also identified that *PART1* could bind to miR- 4516 and inhibit its expression, which is increased after *PART1* knockdown [42]. Another functional study in breast cancer cells demonstrated that *PART1* silencing inhibits cell proliferation, invasion, and migration in MCF7 and T47D cells [43]. These authors also found that *PART1* silencing increased cisplatin sensitivity in MCF7 and T47D breast cancer cells, which was associated with modulation of apoptosis-associated genes, reduced expression of Bcl2, and increased expression of Bax and cleaved caspase-3 [43]. A comprehensive study by Cruickshank et al. (2021) using breast cancer cell lines and PDX models for TNBC and multi-omics analysis demonstrated that *PART1* acts as an oncogene in TNBC and sheds light on the dual action of *PART1* in the tumorigenic process by identifying different coding genes modulated by *PART1* and a landscape of miRNA targets of PART1 that *PART1* could sponge [44]. Taken together, these data indicate that lncRNA *PART1* is a biomarker in breast cancer, particularly in TNBC. It warrants new clinical studies with large cohorts and functional studies to identify and validate new *PART1* targets.

To gain insights into the functional roles of DELncRNAs associated with patient survival, we further analyzed a network of experimentally verified causal interactions among DELncRNAs, proteins, and miRNAs, and then performed functional enrichment analyses. Most of the risk-associated DELncRNAs identified for TNBC (*MIR17HG*, *DGCR5*, and *PART1*) were embedded within a large core and positioned in proximity to classical oncogenic drivers, including MYC, EGFR, KRAS, mTOR, and CTNNB1, as well as tumor suppressors such as TP53 and PTEN. This interconnected hub suggests that DELncRNAs associated with risk are central to oncogenic signaling in proliferative and survival pathways. In contrast, protective DELncRNAs (*PRKCQ-AS1*, *LINC00239*, *VLDLR-AS1*, and *EGOT*) exhibited more restricted and modular connectivity within the network. These DELncRNAs are involved in stress responses and redox regulation, including KEAP1, YBX1, and ABCG2. DGCR5, upregulated in our TNBC cohort, positively influences the activity of the known oncogenic drivers MYC [45], EGFR [46], MTOR [47], and CTNNB1 [45]. Interestingly, MYC and EGFR are also upregulated in our TNBC cohort. In addition, *DGCR5* negatively affects the activity of the tumor suppressor miRNAs mir-195 [48], mir-211-5p [49], and mir-506 [50]. *PART1* was found upregulated in our TNBC cohort. Interestingly*, in vivo* experimental data show that *PART1* can upregulate CTNNB1 by sponging miR-150-5p/miR-520h in colorectal cancer [51]. *MIR17HG*, upregulated in our TNBC cohort, positively influences the activity of *RELA* [52], a known oncogene. Although *RELA* was not found to be upregulated in our TNBC cohort, the progression of TNBCs via this cooperation could be due to a post-translational modification that enhances RELA activity. *PRKCQ-AS1*, upregulated in our TNBC cohort, positively influences the activity of YBX1 [53], a transcription factor that functions primarily as an oncogene. Notably, YBX1 is also upregulated in our TNBC patients. In addition to influencing the activity of YBX1, *PRKCQ-AS1* also negatively influences the activities of miR-1287-5p [53] and miR-545-5p [54], miRNAs that play a role as tumor suppressors in breast cancer. This network suggests that protective DELncRNAs could modulate oxidative stress adaptation and therapy response rather than only proliferative signaling. Importantly, because the network was constructed exclusively from experimentally validated interactions, its topology reflects the current state of documented research. In contrast to TNBC, risk-associated and protective DELncRNAs in non-TNBC are interspersed across both giant component and peripheral subnetworks. Within the large connected component, only MIR17HG and *FAM30A* are present. Together, MIR17HG and FAM30A connect with proteins and miRNAs involved in the coordination of NF-κB/Wnt signaling and epithelial–mesenchymal plasticity. The other risk-associated DELncRNAs present in the network, LY6E-DT and LCAL1, interact with TIMM50 and PRKAA2, which are involved in mitochondrial bioenergetic stress regulation. The protective DELncRNAs in non-TNBC are more numerous and include several DELncRNAs that, in TNBC, occupy relatively central or intermediate positions (e.g., LINC01315, GATA3-AS1, PCAT18, EGOT). In the non-TNBC context, these DELncRNAs show associations with checkpoint regulators and differentiation-related pathways. For example, GATA3-AS1 interacts with canonical tumor suppressors such as TP53 and PTEN, while TP53TG1 interacts with stress-response and MAPK-related mediators, including KRAS. Other protective DELncRNAs participate in miRNA-buffering circuits or smaller regulatory motifs, suggesting a role in stabilizing signaling amplitude rather than amplifying proliferative flux. Future studies using unbiased interaction mapping and functional perturbation will be required to confirm the roles of these DELncRNAs in breast cancer progression.

Although our study is exploratory in nature and aimed at discovering potential new biomarkers, specifically lncRNAs, which play regulatory roles in gene expression and have been linked to the progression of various tumors, including breast cancer [14], our findings may have future applications that impact the management of breast cancer patients. While significant progress has been made in breast cancer prognostic stratification by combining classic clinicopathological parameters with molecular subtype classification based on immunohistochemical markers (ER, PR, HER2, Ki67), additional markers could help refine this stratification and support more personalized therapeutic decisions. In non-TNBC tumors, the expression of lncRNAs, such as *LINC00239*, *LINC01315*, *EGOT*, and *LY6E-DT*, was an independent predictor of survival. If validated, the expression of these lncRNAs, in conjunction with classic markers, could refine the identification of patients who, despite presenting favorable clinical characteristics, are at higher risk of recurrence. In TNBC, an aggressive subtype lacking prognostic markers, the analysis and validation of the lncRNAs *PART*1 and *VLDLR-AS1* (which have proven to be independent markers) could assist in risk stratification of patients with this tumor subtype, who are traditionally treated uniformly. High *PART1* expression and low *VLDLR-AS1* expression could indicate a need for more intensive therapies or more frequent monitoring. Furthermore, the role of lncRNAs such as *PART1*, which modulate the expression of various miRNAs [44], suggests they could serve not only as prognostic biomarkers but also as therapeutic targets or aids in selecting the appropriate treatment regimen.

Our discovery study revealed a set of lncRNAs as candidate biomarkers with the potential to improve prognostic prediction in breast cancer; however, we are aware of the study’s limitations. Firstly, the use of cDNA microarray data with a limited number of lncRNAs. Secondly, our study lacks a complete validation in an independent patient cohort. Third, we are aware that the non-TNBC group encompasses diverse molecular subtypes (Luminal A, Luminal B, and HER2-positive), each with distinct gene expression profiles and clinical courses, and this heterogeneity is a limitation, as it may dilute or mask subtype-specific differences. Therefore, the identified lncRNAs should be considered candidate prognostic biomarkers that require confirmation in external datasets and prospective clinical studies, as well as functional characterization to determine their biological relevance and potential clinical utility.

In conclusion, by reanalyzing cDNA microarray data from women from Latin America with breast cancer, we were able to identify a set of differentially expressed lncRNAs between TNBC and non-TNBC associated with worse survival. Moreover, based on DELncRNA expression, we identified candidate prognostic markers that could predict recurrence risk and overall survival and improve prognostic accuracy in breast cancer patients. These findings provide a basis for future validation and functional studies aimed at determining their biological roles and evaluating their potential as prognostic biomarkers.

## 4. Materials and Methods

### 4.1. Data Collection and cDNA Microarray Analysis

In the present study, we performed a reanalysis of the original microarray data obtained from tumor tissues of 1071 stage II-III breast cancer patients from the Molecular Profile of Breast Cancer Study (MPBCS). This project was an initiative of the US-National Cancer Institute, NIH, USA, to create the United States-Latin America Cancer Research Network (US-LACRN, or LACRN), which involved researchers from Latin American institutions, including Argentina, Brazil, Chile, and Uruguay. The studied population, sample collection and processing, and cDNA microarray analysis have already been described by Llera et al. (2022) [15].

### 4.2. Differential Gene Expression Analyses

Normalized expression data were obtained from Llera et al. (2022) [15], where microarray preprocessing and normalization were performed as described. To identify long non-coding RNAs (lncRNAs) among all probes, we used the HUPO Gene Nomenclature Committee (HGNC) database [55]. We submitted all HGNC symbols previously associated with the probes [15] to the HGNC database and grouped the probes into lncRNAs and mRNAs based on the annotation in the “Locus Type” field. When the description of this field for a given probe was “RNA, long non-coding”, then we classified the probe as lncRNA; if the description was “Gene with protein product”, we classified the probe as mRNA. Probes with other annotations were discarded. Probes corresponding to transcripts annotated as lncRNAs were retained. Probes mapping ambiguously to multiple gene types or lacking updated annotation were excluded. Principal Component Analysis (PCA) was performed using the plotPCA function from the DESeq2 R package to evaluate overall expression patterns and sample clustering. PCA was conducted to assess clustering by intrinsic molecular subtype TNBC vs. non-TNBC, with samples color-coded accordingly. Differential expression analysis between TNBC and non-TNBC samples was performed using the limma package in R. Linear models were fitted using lmFit, and contrasts were defined using makeContrasts. Empirical Bayes moderation was applied using eBayes. Transcripts with a false discovery rate (FDR) *p*-value ≤ 0.05 (Benjamini–Hochberg correction) and absolute log_2_ fold change > 1 were considered differentially expressed. Volcano plots were generated using the ggplot2 package to visualize differentially expressed mRNAs (DEGs) and lncRNAs (DELncRNAs). A heatmap of the top differentially expressed transcripts was generated using the pheatmap package to illustrate expression patterns between TNBC and non-TNBC samples. All analyses were conducted in R (version 4.3.3).

### 4.3. Survival Analyses

Survival analyses were conducted to evaluate the prognostic significance of selected DELncRNAs in TNBC and non-TNBC patients separately. The age of the patients at the time of diagnosis ranged from 22 to 87 years (median 54 years), and the median follow-up was 59 months (range 3 to 87 months). Two clinical endpoints were considered: Disease-Free Survival (DFS) and Overall Survival (OS). Clinical and expression data were merged using patient identifiers. Patients with missing survival information were excluded from the respective analyses. Additionally, for the multivariate Cox regression models, a complete-case analysis (listwise deletion) was applied via the *lifelines* package to address missing clinical covariates. The models were fitted under the standard assumption of proportional hazards for these established clinical covariates. Kaplan–Meier survival curves were generated using the KaplanMeierFitter function from the Python package lifelines (version 0.30.0). Survival differences between groups were assessed using the log-rank test (lifelines.statistics.logrank_test). For gene-level survival stratification, lncRNA expression levels were categorized into high and low groups. Cut-off values were determined by median expression. Univariate Cox proportional hazards regression models were fitted using the CoxPHFitter function from lifelines to estimate hazard ratios (HRs), 95% confidence intervals (CIs), and *p*-values for each lncRNA. Multivariate Cox regression analyses were performed separately for TNBC and non-TNBC patients to evaluate the independent prognostic value of selected lncRNAs while adjusting for relevant clinical variables. Covariates included age (≤50 vs. >50 years), tumor size (≤40 mm vs. >40 mm), clinical stage (Stage I–IIB vs. ≥IIC), and Ki67 status (low vs high, according to St. Gallen classification). Variables were dichotomized prior to modeling as specified above. Hazard ratios, 95% confidence intervals, and corresponding *p*-values were reported. All survival analyses were performed in Python (version 3.12.3).

### 4.4. Expression Validation in the TCGA and METRABIC Databases

To validate the capacity of the identified DELncRNAs to distinguish between TNBC and non-TNBC, transcriptomic data from independent cohorts were retrieved from The Cancer Genome Atlas (TCGA) database and the Molecular Taxonomy of Breast Cancer International Consortium (METABRIC) via cBioPortal. After excluding samples with missing or non-numerical expression data and Normal-like cases, we analyzed validation cohorts comprising 955 TCGA samples and 1708 METABRIC samples. Based on the PAM50 molecular classification, samples were stratified into a TNBC group (consisting of Basal-like cases) and a non-TNBC group (including Luminal A, Luminal B, and HER2-enriched cases). An unsupervised Principal Component Analysis (PCA) was performed on the expression profiles of the 34 lncRNAs present in the TCGA dataset and 21 lncRNAs present in the METABRIC dataset. Before SVD-based PCA execution via the prcomp function in R, expression z-scores were mean-centered and scaled to unit variance. Data manipulation and two-dimensional score plots capturing the highest percentages of total variance (PC1 vs. PC2) were generated using the dplyr and ggplot2 packages, respectively. Supervised hierarchical clustering was conducted to visually explore distinct expression patterns between groups. Heatmaps displaying normalized expression matrices (34 lncRNAs in TCGA and 21 lncRNAs in METABRIC) were constructed using the pheatmap package in R, with samples annotated by their PAM50-derived TNBC or non-TNBC phenotype. Clustering of genes was based on Euclidean distance and the complete linkage algorithm.

### 4.5. Construction of a Causal Interaction Network of DELncRNAs

To investigate how the DELncRNAs associated with DFS and OS in TNBC and non-TNBC patients could potentially and causally cooperate with each other at a molecular systems level to explain their associations with survival features, we built a network containing experimentally proven DELncRNAs-protein and DELncRNAs-miRNAs causal interactions collected from the RNAInter database [56]. We considered only interactions with a confidence score > 0.5. To generate a functional visualization of this network, we used Cytoscape (version 3.10.2) [57].

### 4.6. Functional Enrichment Analysis

To explore the biological functions associated with the causal interaction network of DFS- and OS-associated DELncRNAs, we used the g:Profiler framework [58] implemented in Python (gprofiler-official package version 1.0.0). The list of proteins interacting with the DFS- and OS-associated DELncRNAs was used as the test gene set. All identified proteins found to interact with all 59 DELncRNAs were used as the statistical background. Over-representation analysis was conducted using the default parameters of g:Profiler, including its built-in multiple testing correction method (g:SCS). Enrichment was restricted to Gene Ontology (GO) Biological Process (BP) terms [59,60] to identify significantly overrepresented biological pathways and processes. Terms meeting the default significance threshold of g:Profiler were considered significantly enriched and were used for downstream interpretation.

## Data Availability

The data used in this study will be available from the authors upon request, subject to LACRN’s permission.

## Supplementary Materials

The following supporting information can be downloaded at: https://www.mdpi.com/article/10.3390/ijms27177617/s1.
