# Supplementary material for: Long Noncoding RNAs (lncRNAs) as Biomarkers of Prognosis in TNBC and Non-TNBC Breast Cancer Patients
Source: Int J Mol Sci. 2026 Aug 25;27(17):7617. doi: 10.3390/ijms27177617 (PMC13566564; doi:10.3390/ijms27177617)
Supplement: Supplementary file 1 [file ijms-27-07617-s001.zip › ijms-4412177-supplementary.pdf]

Table S1 - Distribution of demographic and clinicopathological parameters in breast cancer patients with non-TNBC and TNBC tumors

Variable		non-TNBC	TNBC	P value
**Age**	≤ 50 years	281 (35.2%)	83 (48.8%)	0.001 [a]
	> 50 years	518 (64.8%)	87 (51.2%)	
**ER Status**	ER-	103 (13.0%)	140 (82.4%)	< 0.0001 [a]
	ER+	690 (87.0%)	30 (17.6%)	
**PR Status**	PR-	58 (31.7%)	37 (92.5%)	< 0.0001 [a]
	PR+	125 (68.3%)	3 (7.5%)	
**HER2 Status**	HER2 -	124 (73.8%)	35 (92.1%)	0.017 [a]
	HER2 +	44 (26.2%)	3 (7.9%)	
**tumor size**	≤ 4 cm	63 (9.5%)	19 (12.8%)	0.230 [a]
	> 4 cm	598 (90.5%)	129 (87.2%)	
**RCB**	RCB low	55 (24.9%)	18 (26.5%)	0.873 [a]
	RCB high	166 (75.1%)	50 (73.5%)	
**Menopausal status**	premenopausal	229 (32.0%)	65 (43.6%)	0.001[b]
	postmenopausal	477 (66.7%)	78 (52.3%)	
	perimenopausal	9 (1.3%)	6 (4%)	
**First Treatment**	Neoadjuvant Chemotherapy	275 (34.4%)	86 (50.6%)	< 0.0001 [a]
	Primary Surgery	524 (65.6%)	84 (49.4%)	
**RCB**	RCB low	55 (24.9%)	18 (26.5%)	0.873 [a]
	RCB high	166 (75.1%)	50 (73.5%)	

ER – estrogen receptor, PR – progesterone receptor, RCB - Residual Cancer Burden. [a]Chi-square test, [b]Fisher's exact test

Table S2 - Prognostic impact of differentially expressed long non-coding RNAs (DElncRNAs) in triple-negative breast cancer (TNBC).

lncRNA	Disease-free survival		Impact of low expression	Overall survival		Impact of low expression
	HR [95% CI]	P		HR [95% CI]	P	
TNBC						
MIR17HG	0.37 [0.21-0.67]	0.0006	favorable	0.43 [0.23-0.83]	0.0089	favorable
PART1	0.53 [0.30-0.94]	0.0269	favorable	0.45 [0.24-0.86]	0.0128	favorable
LINC01315	0.57 [0.33-1.00]	0.0454	favorable	0.48 [0.25-0.90]	0.0203	favorable
VLDLR-AS1	1.59 [0.91-2.77]	0.0975	-	1.96 [1.04-3.69]	0.0335	unfavorable
PRKCQ-AS1	1.79 [1.03-3.12]	0.0366	unfavorable	1.67 [0.90-3.10]	0.0987	-
DGCR5	0.56 [0.32-0.98]	0.0393	favorable	0.75 [0.41-1.37]	0.3482	-
LINC00239	1.78 [1.02-3.12]	0.0404	unfavorable	1.64 [0.88-3.03]	0.1140	-
EGOT	1.64 [0.94-2.85]	0.0776	-	1.86 [1.00-3.46]	0.0482	unfavorable

Hazard ratios were estimated using Cox proportional hazards regression. High expression was used as reference. Statistical significance was set at $P < 0.05$.

Table S3 - Prognostic impact of differentially expressed long non-coding RNAs (DElncRNAs) in non-triple-negative breast cancer (non-TNBC).

lncRNA	Disease-free survival		Impact of low expression	Overall survival		Impact of low expression
	HR [95% CI]	P		HR [95% CI]	P	
Non-TNBC						
EGOT	1.72 [1.23-2.39]	0.0013	unfavorable	2.54 [1.63-3.96]	0.0000	unfavorable
LINC01124	1.60 [1.15-2.22]	0.0053	unfavorable	1.49 [0.98-2.26]	0.0574	-
LINC00842	1.79 [1.28-2.50]	0.0006	unfavorable	1.38 [0.91-2.09]	0.1249	-
BMPR1B-DT	1.38 [0.99-1.92]	0.0533	-	1.56 [1.02-2.37]	0.0367	unfavorable
SLC25A21-AS1	1.23 [0.88-1.70]	0.2208	-	1.55 [1.02-2.36]	0.0386	unfavorable
SPATA41	1.61 [1.15-2.24]	0.0049	unfavorable	1.77 [1.16-2.71]	0.0076	unfavorable
LINC00239	1.78 [1.02-3.12]	0.0404	unfavorable	1.64 [0.88-3.03]	0.1140	-
FAM30A	0.57 [0.41-0.80]	0.0009	favorable	0.55 [0.36-0.83]	0.0043	favorable
PCAT18	1.47 [1.06-2.05]	0.0202	unfavorable	1.46 [0.97-2.22]	0.0712	-
LCAL1	0.69 [0.49-0.96]	0.0247	favorable	0.86 [0.43-0.99]	0.0450	favorable
LINC01315	1.63 [1.17-2.27]	0.0037	unfavorable	1.45 [0.96-2.20]	0.0741	-
LINC00908	1.61 [1.15-2.24]	0.0048	unfavorable	2.12 [1.37-3.27]	0.0005	unfavorable
LINC00683	1.32 [0.99-1.83]	0.0985	-	1.83 [1.19-2.80]	0.0050	unfavorable
LINC01127	0.86 [0.62-1.20]	0.3789	-	0.66 [0.44-1.00]	0.0464	favorable
MIR29B2CHG	1.12 [0.81-1.55]	0.4951	-	1.53 [1.01-2.32]	0.0447	unfavorable
LINC01644	1.34 [0.97-1.86]	0.0791	-	1.73 [1.14-2.64]	0.0098	unfavorable
TP53TG1	1.40 [1.01-1.95]	0.0425	unfavorable	1.65 [1.08-2.51]	0.0186	-
FUT8-AS1	1.48 [1.06-2.06]	0.0197	unfavorable	1.55 [1.02-2.35]	0.0391	unfavorable
MIR1915HG	0.72 [0.51-1.00]	0.0463	favorable	0.77 [0.51-1.17]	0.2214	-
MIR3936HG	1.40 [1.01-1.95]	0.0439	unfavorable	1.48 [0.98-2.24]	0.0634	-
MIR17HG	0.69 [0.50-0.96]	0.0286	favorable	0.89 [0.59-1.34]	0.5771	-
LY6E-DT	0.74 [0.53-1.03]	0.0753	-	0.64 [0.42-0.97]	0.0351	favorable
LINC01152	1.19 [0.86-1.65]	0.3003	-	1.53 [1.01-2.31]	0.0438	unfavorable
ZNF205-AS1	1.65 [1.18-2.28]	0.0032	unfavorable	2.12 [1.38-3.26]	0.0005	unfavorable
IDI2-AS1	0.62 [0.45-0.87]	0.0048	favorable	0.65 [0.43-0.99]	0.0437	favorable
CT62	1.46 [1.05-2.03]	0.0234	unfavorable	1.34 [0.89-2.03]	0.1574	-
GATA3-AS1	1.43 [1.03-1.98]	0.0335	unfavorable	1.75 [1.15-2.67]	0.0084	unfavorable

Hazard ratios were estimated using Cox proportional hazards regression. High expression was used as reference. Statistical significance was set at $P < 0.05$.

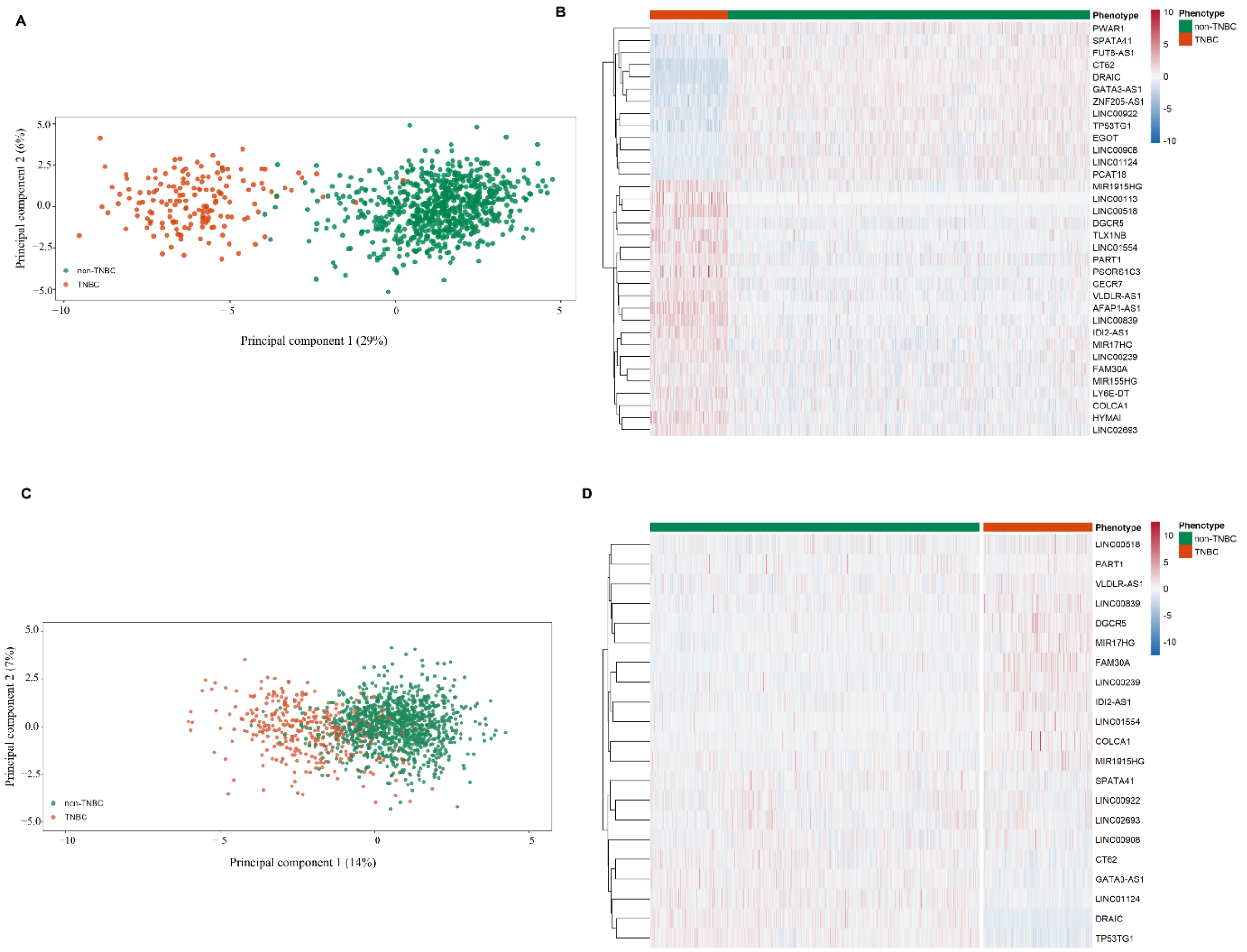

Figure S1. Principal component analysis (PCA) and differential expression profile of the DELncRNA signature in TCGA and METABRIC cohorts. (A) Principal component analysis (PCA) of the 34 lncRNAs available in TCGA breast cancer samples. PCA using normalized expression data from 955 breast cancer samples was used to assess transcriptomic variation between TNBC (n=163) and non-TNBC (n=792) groups. (B) Heatmap of the 34 differentially expressed lncRNAs based on hierarchical clustering of normalized expression values in the TCGA cohort. Columns represent individual samples, rows represent lncRNAs, and samples are grouped according to TNBC status. (C) PCA plot illustrating transcriptomic variation and sample segregation between TNBC and non-TNBC groups in the METABRIC validation cohort using the 21 lncRNA available. (D) Heatmap of the 21 lncRNAs displaying hierarchical clustering and relative expression levels across METABRIC samples grouped by TNBC phenotype. Each point in PCA plots represents a sample colored by subtype (TNBC, orange; non-TNBC, green), and axis percentages indicate variance explained by the first two principal components. The heatmap color scale reflects relative expression levels (Z-score normalized).

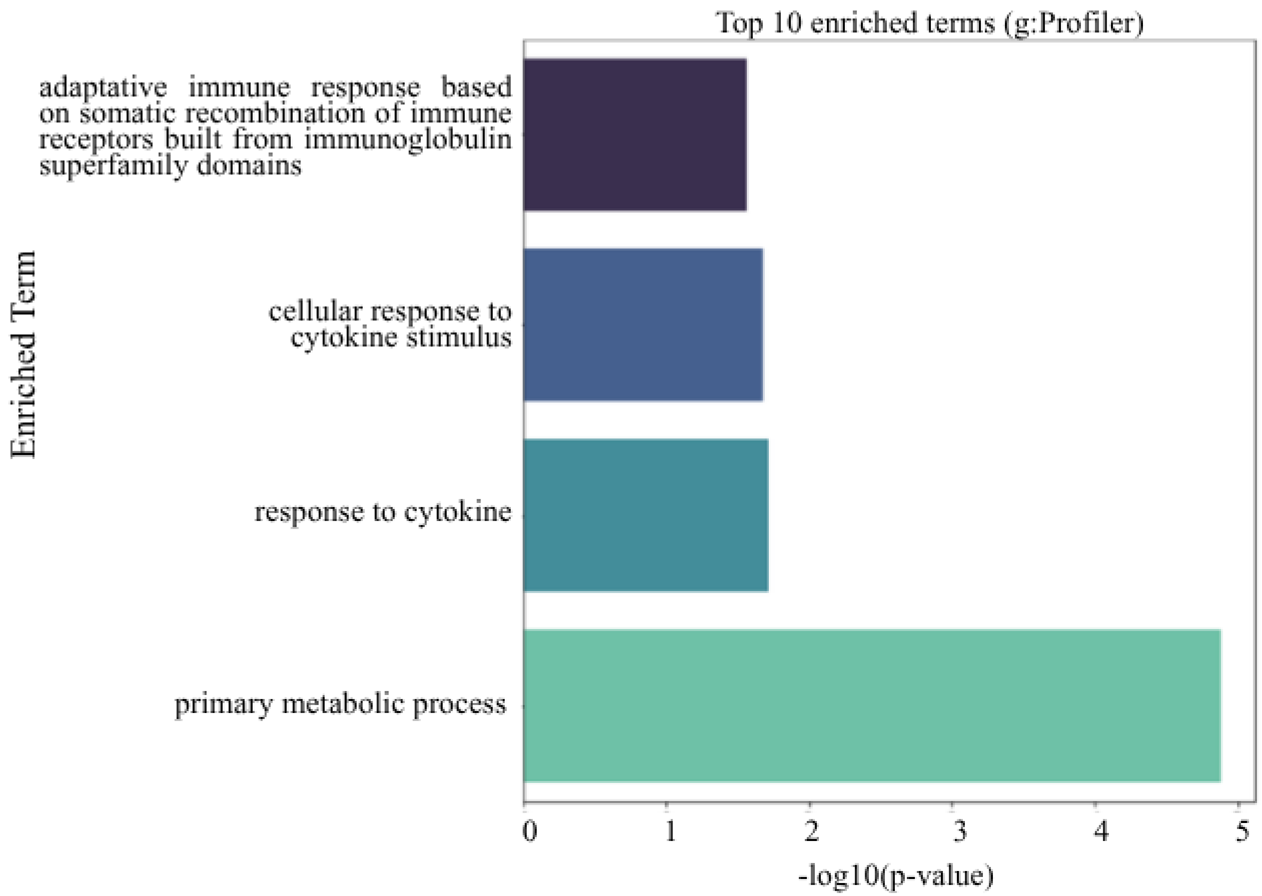

Figure S2 Functional enrichment analysis of DFS- and OS-associated DELncRNAs. Gene Ontology (GO) Biological Process enrichment analysis of proteins interacting with the survival-associated DELs. Bars represent significantly enriched GO terms. These terms are ranked by statistical significance and displayed as $-\log_{10}$(P-value).
